# Sequence-Based Polymorphisms in the Mitochondrial D-Loop and Potential SNP Predictors for Chronic Dialysis

**DOI:** 10.1371/journal.pone.0041125

**Published:** 2012-07-18

**Authors:** Jin-Bor Chen, Yi-Hsin Yang, Wen-Chin Lee, Chia-Wei Liou, Tsu-Kung Lin, Yueh-Hua Chung, Li-Yeh Chuang, Cheng-Hong Yang, Hsueh-Wei Chang

**Affiliations:** 1 Division of Nephrology, Department of Internal Medicine, Mitochondrial Research Unit, Kaohsiung Chang Gung Memorial Hospital, Chang Gung University College of Medicine, Kaohsiung, Taiwan; 2 School of Pharmacy, Kaohsiung Medical University, Kaohsiung, Taiwan; 3 Department of Neurology and Mitochondrial Research Unit, Kaohsiung Chang Gung Memorial Hospital and Chang Gung University College of Medicine, Kaohsiung, Taiwan; 4 Institute of Biomedical Sciences, National Sun Yat-Sen University, Kaohsiung, Taiwan; 5 Department of Chemical Engineering & Institute of Biotechnology and Chemical Engineering, I-Shou University, Kaohsiung, Taiwan; 6 Department of Electronic Engineering, National Kaohsiung University of Applied Sciences, Kaohsiung, Taiwan; 7 Department of Biomedical Science and Environmental Biology, Kaohsiung Medical University, Taiwan; 8 Center of Excellence for Environmental Medicine, Cancer Center, Kaohsiung Medical University Hospital, Kaohsiung Medical University, Kaohsiung, Taiwan; Centers for Disease Control and Prevention, United States of America

## Abstract

**Background:**

The mitochondrial (mt) displacement loop (D-loop) is known to accumulate structural alterations and mutations. The aim of this study was to investigate the prevalence of single nucleotide polymorphisms (SNPs) within the D-loop among chronic dialysis patients and healthy controls.

**Methodology and Principal Findings:**

We enrolled 193 chronic dialysis patients and 704 healthy controls. SNPs were identified by large scale D-loop sequencing and bioinformatic analysis. Chronic dialysis patients had lower body mass index, blood thiols, and cholesterol levels than controls. A total of 77 SNPs matched with the positions in reference of the Revised Cambridge Reference Sequence (CRS) were found in the study population. Chronic dialysis patients had a significantly higher incidence of 9 SNPs compared to controls. These include SNP5 (16108Y), SNP17 (16172Y), SNP21 (16223Y), SNP34 (16274R), SNP35 (16278Y), SNP55 (16463R), SNP56 (16519Y), SNP64 (185R), and SNP65 (189R) in D-loop of CRS. Among these SNPs with genotypes, SNP55-G, SNP56-C, and SNP64-A were 4.78, 1.47, and 5.15 times more frequent in dialysis patients compared to controls (*P*<0.05), respectively. When adjusting the covariates of demographics and comorbidities, SNP64-A was 5.13 times more frequent in dialysis patients compared to controls (*P*<0.01). Furthermore, SNP64-A was found to be 35.80, 3.48, 4.69, 5,55, and 4.67 times higher in female patients and in patients without diabetes, coronary artery disease, smoking, and hypertension in an independent significance manner (*P*<0.05), respectively. In patients older than 50 years or with hypertension, SNP34-A and SNP17-C were found to be 7.97 and 3.71 times more frequent (*P*<0.05) compared to patients younger than 50 years or those without hypertension, respectively.

**Conclusions and Significance:**

The results of large-scale sequencing suggest that specific SNPs in the mtDNA D-loop are significantly associated with chronic dialysis. These SNPs can be considered as potential predictors for chronic dialysis.

## Introduction

Mitochondria (mt) are organelles that are susceptible to oxidative stress. The presence of excessive amounts of reactive oxidative species (ROS) results in mitochondrial oxidative damage and inefficient repair of mtDNA [1]–[3]. This can contribute to pathophysiological processes, including aging, degenerative disease [4]–[6] and cancer [7]. In these circumstances, somatic mutations are also generated [8].

The displacement loop (D-loop) regions of mtDNA does not encode any functional proteins [9], [10] and is known to accumulate mutations at a higher frequency than other regions of mtDNA in the setting of increased oxidative stress [11]. The D-loop contains the initial site of heavy chain replication and the promoters for heavy and light chain transcription. Therefore, it is responsible for the regulation of mtDNA replication and transcription [10], [11]. The D-loop is highly polymorphic, and some polymorphisms are associated with aging [12]–[15], coronary artery disease [16], and a variety of tumors, including lung [17], colorectal [18], liver [19], gastric [20], breast [21], cervical [22], melanoma [23], head and neck [24], oral [25], and kidney [26] cancers. However, D-loop polymorphisms are not associated with prostate cancer 27,28. Most of these D-loop studies focus on some cancer-associated single nucleotide polymorphisms (SNPs) for mtDNA, which were accompanied by poly-C tract alterations [21], [24], [25], [29], [30]. However, D-loop polymorphisms have not been systematically characterized in chronic dialysis patients.

Complications of chronic kidney disease (CKD) promote morbidity and mortality [31]. CKD patients can be classified according to kidney function along a continuum from mild renal dysfunction to irreversible kidney failure. CKD increases oxidative stress [32] which has been demonstrated to influence mtDNA content in CKD patients [33], [34].

Because the D-loop region susceptible to oxidative stress, we hypothesized that specific SNP patterns in the D-loop of chronic dialysis patients may serve as potential genetic markers for chronic dialysis. To examine this hypothesis, we performed D-loop sequencing and used bioinformatic tools to identify SNPs that were associated with chronic dialysis when compared to healthy controls.

**Table 1:** Basic demographic characteristics of patients and controls.

			patients		controls		Chi-square
		total	n	%	n	%	*P* value
Total		897	193	21.5	704	78.5	
Sex	female	427	115	59.6	312	44.3	0.0002
	male	470	78	40.4	392	55.7	
Age	≤50	422	97	50.3	325	46.2	0.3127
	>50	475	96	49.7	379	53.8	
	Mean (SD)		49.0	(13.9)	51.9	(12.9)	0.0055
DM	N	836	161	83.4	675	98.3	<0.0001
	Y	44	32	16.6	12	1.7	
CHD	N	854	170	88.1	684	98.4	<0.0001
	Y	34	23	11.9	11	1.6	
HT	N	593	109	56.5	484	69.6	0.0006
	Y	295	84	43.5	211	30.4	
Smoke	N	698	170	88.1	528	75.0	0.0001
	Y	199	23	11.9	176	25.0	
BMI	Mean (SD)		22.3	(3.8)	24.5	(3.5)	<0.0001
TBARS	Mean (SD)		1.1	(0.6)	1.2	(0.8)	0.0801
Thiols	Mean (SD)		1.5	(0.5)	2.0	(0.4)	<0.0001
TG	Mean (SD)		169.5	(128.1)	130.4	(85.7)	<0.0001
Chol	Mean (SD)		189.4	(35.7)	202.1	(37.9)	<0.0001

Abbreviations and/or units: CHD: coronary heart disease, HT: hypertension, BMI: body mass index, TBARS: thiobarbituric acid reactive substance (µM), Thiols (µM); TG: triglyceride (mg/dL), Chol: cholesterol (mg/dL).

## Materials and Methods

### Subjects

We enrolled 704 unrelated Taiwanese of ethnic Chinese background in this study through the hospital health examination center after giving consent. Participants included 312 men and 392 women with a mean age of 51.9 years. We enrolled 193 dialysis patients from the outpatient dialysis unit of the same hospital. They were composed of 78 men and 115 women with a mean age of 49 years. Venous blood samples were collected after overnight fasting. The serum was separated using a centrifuge and stored at −80°C. DNA was isolated from leucocytes using PUREGENE® DNA Purification kit (Gentra, Minneapolis, MN, USA) and stored at −20°C. The protocol for the present study was approved by the Committee on Human Research at Kaohsiung Chang Gung Memorial Hospital (CMRPG850271, CMRPG850272, CMRPG850242, CMRPG850252, IRB 95-0395B) and conducted in accordance with the Declaration of Helsinki. All participants signed a written informed consent form to obtain the approval for participation in this study.

### Assessment of Oxidative and Anti-oxidative Stress Capacities

Serum free thiols were determined by direct reaction of the thiols with 5,5-dithiobis(2-nitrobenzoic acid) (DTNB) to form 5-thio-2-nitrobenzoic acid (TNB). The amount of thiols was calculated from the absorbance determined using the extinction coefficient of TNB (A412 = 13,600 M^−1^ cm^−1^). The serum thiobarbituric acid reactive substance (TBARS) concentration was assessed according to the method of Ohkawa *et al.* [35]. Results are expressed as micromoles of TBARS per liter. A standard curve of TBARS was obtained by hydrolysis of 1,1,3,3-tetraethoxypropane (TEPP).

**Table 2:** SNP identification from aligned sequences of cases and controls and their positional information.

SNP No.	Align-position*1	D-loop position*2	IUPAC code	SNP No.	Align-position*1	D-loop position*2	IUPAC code
**1**	51	16051	R	**40**	298	16298	Y
**2**	86	16086	Y	**41**	304	16304	Y
**3**	92	16092	H	**42**	309	16309	R
**4**	93	16093	Y	**43**	311	16311	Y
**5**	108	16108	Y	**44**	316	16316	R
**6**	111	16111	Y	**45**	319	16319	R
**7**	126	16126	Y	**46**	324	16324	Y
**8**	129	16129	R	**47**	327	16327	Y
**9**	136	16136	Y	**48**	335	16335	R
**10**	140	16140	Y	**49**	355	16355	Y
**11**	145	16145	R	**50**	356	16356	Y
**12**	148	16148	Y	**51**	357	16357	Y
**13**	157	16157	Y	**52**	362	16362	Y
**14**	162	16162	R	**53**	390	16390	R
**15**	164	16164	R	**54**	399	16399	R
**16**	167	16167	Y	**55**	463	16463	R
**17**	172	16172	Y	**56**	519	16519	Y
**18**	209	16209	Y	**57**	662	93	R
**19**	217	16217	Y	**58**	672	103	R
**20**	218	16218	Y	**59**	715	146	H
**21**	223	16223	Y	**60**	719	150	Y
**22**	227	16227	R	**61**	720	151	Y
**23**	234	16234	Y	**62**	721	152	Y
**24**	235	16235	R	**63**	722	153	R
**25**	243	16243	H	**64**	754	185	R
**26**	248	16248	Y	**65**	758	189	R
**27**	249	16249	Y	**66**	763	194	Y
**28**	256	16256	Y	**67**	764	195	Y
**29**	257	16257	H	**68**	768	199	Y
**30**	260	16260	Y	**69**	769	200	R
**31**	261	16261	Y	**70**	773	204	Y
**32**	266	16266	N	**71**	776	207	R
**33**	272	16272	R	**72**	779	210	R
**34**	274	16274	R	**73**	786	217	Y
**35**	278	16278	Y	**74**	803	234	R
**36**	290	16290	Y	**75**	804	235	R
**37**	291	16291	Y	**76**	885	317	Y
**38**	295	16295	Y	**77**	1019	461	Y
**39**	297	16297	Y				

* 1.The positions are defined by the aligned sequences from cases and controls. Due to the poor quality at both 5′ and 3′ ends for PCR amplified by primers L15911/H602 as described in materials and methods, the sequences of nt15911–16000 and nt486–602 of the NC_012920 were excluded. nt249/353/354 of the NC_012920 were not included because they were not found in our sequencing data.

* 2.The position for the D-loop in the Revised Cambridge Reference Sequence (“rCRS”; NC_012920).

### D-loop Sequencing

The mtDNA control region segment (relative to nucleotide (nt) regions 15911–16569 and 1–602 in the Revised Cambridge Reference Sequence (“rCRS”) [36]; NC_012920) was amplified using the forward primer L15911 (5′-ACCAGTCTTGTAAACCGGAG-3′) and the reverse primer H602 (5′-GCTTTGAGGAGGTAAGCTAC-3′). The products were purified with gel extraction kits (Watson BioMedicals Inc.) and sequenced using primer L15911 and primer L29 (5′-CTCACGGGAGCTCTCCATGC-3′) on an ABI 377XL DNA Sequencer (Applied Biosystems, Foster, CA, USA). However, due to the conversion of thymine to cytosine and the presence of homopolymeric cytosine tracts at nt16184–16193 and nt303–315 within the D-loop region of some subjects, the sequencing procedure was prematurely terminated. Therefore, we also performed reverse sequencing using 2 additional sets of primers, H81 (5′-CAGCGTCTCGCAATGCTATC-3′) and H528 (5′-TTCGGGGTATGGGGTTAGCA-3′). The polymerase chain reaction (PCR) conditions used were as follows: an initial denaturation step at 95°C for 5 min, followed by 35 cycles of denaturation at 95°C for 1 min, annealing at 60°C for 1 min, and extension at 68°C for 2 min, with a final extension of 10 min at 72°C. The PCR fragments were analyzed by electrophoresis on a 2% agarose gel and visualized by staining with ethidium bromide.

**Table 3:** The 9 SNPs with significantly different genotype distributions between patients and controls.

				patients		controls		Chi-square
Variable* ^1^	Variable* ^2^		total	n	%	n	%	*P* value
total			897	193		704		
SNP 5	16108Y	C	877	185	95.9	692	98.3	0.0419
		T	20	8	4.1	12	1.7	
SNP 17	16172Y	C	126	36	18.7	90	12.8	0.0376
		T	771	157	81.3	614	87.2	
SNP 21	16223Y	C	394	97	50.3	297	42.2	0.0453
		T	503	96	49.7	407	57.8	
SNP 34	16274R	A	13	6	3.1	7	1.0	0.0294
		G	884	187	96.9	697	99.0	
SNP 35	16278Y	C	843	188	97.4	655	93.0	0.0238
		T	54	5	2.6	49	7.0	
SNP 55	16463R	A	888	188	97.4	700	99.4	0.0125
		G	9	5	2.6	4	0.6	
SNP 56	16519Y	C	488	119	61.7	369	52.4	0.0224
		T	409	74	38.3	335	47.6	
SNP 64	185R	A	25	14	7.3	11	1.6	0.0000
		G	872	179	92.7	693	98.4	
SNP 65	189R	A	874	184	95.3	690	98.0	0.0373
		G	23	9	4.7	14	2.0	

* 1The annotation of these SNPs is listed in Table 2.

* 2SNPs in rCRS position with IUPAC code.

### SNP Identification

DNA sequences were analyzed by using the DNASTAR software and Bio Edit Sequence Alignment Editor freeware (http://www.mbio.ncsu.edu/bioedit/bioedit.html). After multiple sequence alignments were performed, both 5′ and 3′ ends of the sequences were trimmed into blunt ends. The SNPs were identified by calculating each nucleotide (A, T, C, or G) for each position in the trimmed and aligned sequences by “count if = ” in Excel software. SNP frequencies greater than 1% were selected for further investigation. The SNPs were compared to the D-loop polymorphisms in rCRS as shown in MITOMAP [37] (http://www.mitomap.org/MITOMAP/PolymorphismsControl).

**Table 4:** The OR and AOR for the 3 SNPs selected by backward logistic regression.

Variable* ^1^	OR* ^2^	95% CI	*P* value	AOR* ^3^	95% CI	*P* value
SNP 55 G *vs.* A	4.78	1.26–18.09	0.0212	1.35	0.15–12.41	0.7886
SNP 56 C *vs.* T	1.47	1.06–2.04	0.0225	1.41	0.89–2.24	0.1441
SNP 64 A *vs.* G	5.15	2.29–11.60	0.0001	5.13	1.61–16.35	0.0057

* 1.The annotation of these SNPs is listed in Table 2.

* 2.Odds ratios (ORs) were computed by having only SNP variables in the logistic regression.

* 3.Adjusted odds ratios (AORs) were computed by having SNP variables in the analysis model with covariates of sex, diabetes mellitus, coronary heart disease, smoker, hypertension, age, body mass index, thiobarbituric acid reactive substance, thiols, triglyceride, and cholesterol.

**Table 5:** The OR and AOR for the 9 SNPs selected by backward logistic regression for subgroups related to several basic demographic characteristics.

(no adjust) *1	female			male			age< = 50			age>50					
effect *2	*OR*	95% CI	*P*	*OR*	95% CI	*P*	*OR*	95% CI	*P*	*OR*	95% CI	*P*			
SNP 5 T *vs.* C				5.88	1.78–19.44	**0.004**									
SNP 17 C *vs.* T							1.81	1.01–3.28	**0.048**						
SNP 21 C *vs.* T				2.06	1.22–3.47	**0.007**									
SNP 34 A *vs.* G										5.26	1.15–24.00	**0.032**			
SNP 35 C *vs.* T															
SNP 55 G *vs.* A	6.10	1.10–33.79	**0.039**				11.43	1.17–111.50	**0.036**						
SNP 56 C *vs.* T															
SNP 64 A *vs.* G	15.24	3.29–70.73	**0.001**							5.59	1.89–16.57	**0.002**			
SNP 65 G *vs.* A				8.14	1.57–42.28	**0.013**									

(no adjust) *1	no DM			no CHD			non smoker			no HT			having HT		
effect *2	*OR*	95% CI	*P*	*OR*	95% CI	*P*	*OR*	95% CI	*P*	*OR*	95% CI	*P*	*OR*	95% CI	*P*
SNP 5 T *vs.* C							2.81	1.09–7.25	**0.033**						
SNP 17 C *vs.* T													3.10	1.48–6.51	**0.003**
SNP 21 C *vs.* T															
SNP 34 A *vs.* G	3.20	1.02–10.00	0.046												
SNP 35 C *vs.* T				3.97	1.22–12.91	**0.022**				4.62	1.10–19.41	**0.037**			
SNP 55 G *vs.* A	4.59	1.14–18.58	**0.033**	5.14	1.36–19.37	**0.016**	5.85	1.38–24.77	**0.016**	6.06	1.33–27.54	**0.020**			
SNP 56 C *vs.* T															
SNP 64 A *vs.* G	4.13	1.66–10.23	**0.002**	4.52	1.89–10.85	**0.001**	7.02	2.59–19.02	**0.000**	5.20	1.84–14.68	**0.002**			
SNP 65 G *vs.* A													4.46	1.05–18.98	**0.043**

(Adjust) *3	female			male			age< = 50			age>50					
effect *4	*AOR*	95% CI	*P*	*AOR*	95% CI	*P*	*AOR*	95% CI	*P*	*AOR*	95% CI	*P*			
SNP 5 T *vs.* C				4.53	0.80–25.65	0.088									
SNP 17 C *vs.* T							1.48	0.55–3.96	0.435						
SNP 21 C *vs.* T				2.05	0.95–4.40	0.067									
SNP 34 A *vs.* G										7.97	1.25–50.94	**0.028**			
SNP 35 C *vs.* T															
SNP 55 G *vs.* A	0.97	0.02–41.98	0.986				14.39	0.30–685.42	0.176						
SNP 56 C *vs.* T															
SNP 64 A *vs.* G	35.80	3.23–396.84	**0.004**							3.67	0.78–17.25	0.100			
SNP 65 G *vs.* A				4.84	0.35–67.24	0.240									

(Adjust) *3	no DM			no CHD			non smoker			no HT			having HT		
effect *4	*AOR*	95% CI	*P*	*AOR*	95% CI	*P*	*AOR*	95% CI	*P*	*AOR*	95% CI	*P*	*AOR*	95% CI	*P*
SNP 5 T *vs.* C							3.02	0.80–11.42	0.102						
SNP 17 C *vs.* T													3.71	1.10–12.55	**0.035**
SNP 21 C *vs.* T															
SNP 34 A *vs.* G	4.53	1.02–20.21	0.048												
SNP 35 C *vs.* T				3.76	0.84–16.81	0.083				5.95	0.73–48.65	0.096			
SNP 55 G *vs.* A	1.55	0.15–15.83	0.713	1.45	0.16–13.54	0.742	2.06	0.16–27.11	0.582	1.97	0.16–24.08	0.595			
SNP 56 C *vs.* T															
SNP 64 A *vs.* G	3.48	0.97–12.55	0.056	4.69	1.44–15.27	**0.010**	5.55	1.38–22.28	**0.016**	4.67	1.02–21.24	**0.046**			
SNP 65 G *vs.* A													4.19	0.41–42.36	0.225

* 1.Odds ratios (ORs) were computed by having only SNP variables in the logistic regression.

* 2.Significant SNPs were selected by backward logistic regression for subgroups.

* 3.Adjusted odds ratios (AORs) were computed by having SNP variables in the analysis model with covariates of sex, diabetes mellitus, coronary heart disease, smoker, hypertension, age, body mass index, thiobarbituric acid reactive substance, thiols, triglyceride, and cholesterol.

* 4.Adjusted covariates were added in models with significant SNPs.

*P* = *P* value.

## Statistical Analysis

Chi-square tests were used to compare basic characteristics between patients and controls. A sequence of analyses was adopted for SNP selection. The Chi-square tests were first used to compare distributions of SNPs between patients and controls. Nine SNPs with significant differences and with sufficient cell sizes were chosen for further analysis. These 9 SNPs were included in a logistic regression model with backward selection. Only statistically significant SNPs were selected by logistic regression. The same logistic regression selection process was also conducted for several subgroups. Lastly, the adjusted odds ratios (AOR) from selected SNPs were computed on the basis of logistic regression with additional covariates of basic demographic characteristics (Table 1). The statistical data were expressed as mean ± SD. A *P* value of less than 0.05 was considered as statistically significant.

## Results

### Basic Demographic Characteristics

The study participants included 193 dialysis patients and 704 healthy controls, and their basic characteristics are shown in Table 1. Most of these characteristics were found to be significantly different, except for age groups and blood TBARS levels. The patients were 3 years younger (49.0±13.9 *vs.* 51.9±12.9) than the controls and had lower values of body mass index (BMI), blood thiols, and cholesterol levels. The mean triglyceride (TG) level was higher in patients than in controls. There was a significantly higher incidence of comorbidities of diabetes, hypertension (HT), and coronary heart disease (CHD) in dialysis patients compared to controls.

### D-loop Sequencing, Alignment, and SNP Identification

There are 2 poly-C regions in the mitochondrial D-loop that stretch between nt16180–16195 [38] and nt303–315 [9]. Because the length of these mononucleotide repeats varies, they may interfere the sequence alignment processing or lead to error alignment in part. Accordingly, the sequences for these 2 repeat regions were replaced with the corresponding sequences for the reference CRS to improve the performance of sequence alignment. The sequencing data from the 5′ and 3′ ends of nt15911–16000 and nt486–602 were of poor quality and, therefore, were trimmed after confirmation of sequence alignment. Finally, aligned sequences were trimmed to the same length ranging from nt16000–16569 and nt1–485 for further SNP identification (Table S1 and Table S2; all D-loop trimmed sequences for cases and controls and their alignment visualization, respectively). After examining each nt for each position of the trimmed sequence, 77 SNPs with frequencies greater than 1% were identified (Table S3). The relationships between positions of the aligned sequences and D-loop in the reference CRS as well as the SNP types in the IUPAC code are listed in Table 2.

### Significance Analysis for 77 Individual SNPs

The *P* values for 77 individual SNPs with A, G, C, and T distribution data were analyzed (Table S4). Nine SNPs were selected from 77 SNPs by Chi-square tests with significant differences and sufficient cell sizes; their genotype distributions are compared in Table 3. For each SNP, the genotype that appeared at a higher frequency in patients was selected as the indicator. Hence, the indicators for the SNPs 5, 17, 21, 34, 35, 55, 56, 64, and 65 (16108Y, 16172Y, 16223Y, 16274R, 16278Y, 16463R, 16519Y, 185R, and 189R) were T, C, C, A, C, G, C, A, and G, respectively. These 9 indicators were further added into a logistic regression by employing the backward selection method.

### Backward Logistic Regression Analysis for 9 SNPs

As shown in Table 4, we identified 3 statistically significant indicators (SNP55 G, SNP56 C, and SNP64 A). Individuals with the SNP55 G increase risk of chronic dialysis by 4.78 times (OR, 95% CI = 1.26∼18.09, *P* = 0.0212). SNP56 C or SNP64 A subjects increase risk of chronic dialysis by 1.47 (95% CI = 1.06∼2.04, *P* = 0.0225) or 5.15 (95% CI = 2.29∼11.60, *P* = 0.0001) times. The AORs of the 3 SNPs were further computed by adding the covariates shown in Table 1 into the logistic regression analysis. Following this, only SNP64 A remained significant (OR = 5.13, 95% CI = 1.61∼16.35, *P* = 0.0057). Hence, SNP64 is only an independent SNP for disease as well as for the patients’ basic characteristics. On the other hand, while SNP55 and SNP56 found in the backward logistic regression could only be considered as independent SNPs among the 77 SNPs, they were affected by covariates.

### Stepwise Regression for Subgroups Related to Several Basic Demographic Characteristics

Similar procedures were also conducted in several subgroups (Table 5). While the frequencies of SNP55 and SNP64 were found to be significantly higher in women, only those with SNP64 A genotype had a statistically significant higher risk of chronic dialysis (AOR = 35.80, 95% CI = 3.23∼396.84, *P* = 0.004). In subjects older than 50 years, SNP34 A genotype was significantly associated with chronic dialysis (AOR = 7.97, 95% CI = 1.25∼50.94, *P* = 0.028). For subjects without diabetes, without CHD, no smoking habit, or without HT, SNP64 A was the independent SNP in association with chronic dialysis (AOR = 3.48, 4.69, 5.55, and 4.67, *P* = 0.010, 0.016, and 0.046, respectively). For subjects with history of hypertension, SNP17 C was significantly associated with chronic dialysis (AOR = 3.71, 95% CI = 1.10∼12.55, *P* = 0.035).

## Discussion

To date, most association studies of chronic dialysis focus on the nuclear genome [39]–[43] rather on mtDNA. In our previous report [9], we addressed the association between polymorphisms in the poly-C tract (D310) of the mtDNA D-loop and probability of dialysis treatment. However, we found that the poly-C tract was not significantly different in dialysis patients compared with healthy controls. In addition to the poly-C tract, SNPs are also found in the D-loop. Therefore, we decided to determine whether there was any association between chronic dialysis and SNPs in the D-loop in this study.

Using sequence alignment, we found 9 SNPs present at significantly higher frequency in dialysis patients (SNP5, 17, 21, 34, 35, 55, 56, 64, and 65). Among them, 3 significant indicators (SNP55 G, SNP56 C, and SNP64 A) were independently associated with a high risk of chronic dialysis. Furthermore, only women with the SNP64 A genotype were statistically significant to be associated with chronic dialysis. SNP34 A was significantly associated with chronic dialysis in subjects older than 50 years. For subjects without diabetes, CHD, or hypertension, or in non-smokers, SNP64 A was statistically associated with chronic dialysis. Individuals with history of hypertension were significantly associated with chronic dialysis if they carried SNP17 C.

In this study, we focused solely on the question of whether individual SNPs within the D-loop were associated with chronic dialysis. However, the consideration of interdependence among SNPs was found to improve the association of genetic variations with several diseases [44], [45] and cancers [46]–[54]. Therefore, we cannot exclude the possibility that some rare SNPs may still contribute to the synergistic association with chronic dialysis.

According to the diseases-associated mtSNPs in the D-loop locus in MITOMAP [37] (http://www.mitomap.org/bin/view.pl/MITOMAP/MutationsCodingControl), only 7 mtSNPs were reported. With reference to the rCRS, these are C114T, C150T, T195C, C309CC, T16189C, A16300G, and C16519T. We only identified C150T (SNP60), T195C (SNP67), and C16519T (SNP56) in our study (Table 2), and of these, only C16519T (SNP56) was significantly associated with chronic dialysis (Table 3 and Table 4). Similarly, C16519T was reported to be associated with “cyclic vomiting syndrome with migraine” [55], [56]. When stratification of genotypes by demographic characteristics was considered, C16519T did not appear to be a marker associated with chronic dialysis (Table 5). On the contrary, we identified several novel mtSNPs associated with chronic dialysis, suggesting that these mtSNPs are potential genetic markers for this disease.

The acquisition of ROS-induced mutations in CKD may be a consequence of increased oxidative burden in patients with chronic renal failure [9], [32], [33], [57]. For example, elevated oxidative stress in chronic peritoneal dialysis patients may lead to alterations in the mtDNA copy number in peripheral leukocytes [33]. In our current study, the mtSNPs listed in Table 3 were homoplasmic, as revealed by sequencing chromatograms (data not shown) [58]–[60]. However, we cannot exclude the possibility that a minor fraction of heteroplasmic mutations, below the level of sensitivity of the sequencing method that we used, may be present. We suggest that additional PCR/restriction fragment length polymorphism (RFLP) analysis may assist in the identification of mitochondrial heteroplasmy [61], [62]. In light of this, we are unable to identify mtSNPs that are suitable as progression markers for CKD with our current data, since our sequencing method lacked sufficient sensitivity to detect ROS-induced mutations. Therefore, the biological and clinical significance of the homoplasmic mtSNPs are more suitable as potential genetic markers for chronic dialysis, rather than progression markers of CKD.

To the best of our knowledge, this is the first report of SNPs in the mtDNA D-loop showing that they are significantly associated with chronic dialysis. The study also demonstrated the relationship of SNPs with comorbidities in dialysis patients. One may postulate that the presence of these SNPs is a risk factor for the development of end-stage renal disease, and that they may be used as markers to predict the likelihood of dialysis. In the future, further studies are needed to establish the role of these SNPs in the pathophysiology of CKD and to validate their clinical application.

## Supporting Information

Table S1 **Case (n = 193)-D-loop trimmed sequences in FSATA format.**
(TXT)

Table S2 **Control (n = 704)-D-loop trimmed sequences in FSATA format.**
(TXT)

Table S3 **77 SNP genotype raw data for cases and controls.**
(XLSX)

Table S4 ***P*** **values of 77 individual SNPs for cases and controls.**
(XLSX)
